# Anomalous Coronary Arteries: A Cause for Malignant Arrhythmias

**DOI:** 10.7759/cureus.39658

**Published:** 2023-05-29

**Authors:** Atif AlQubbany, Yazeed Alqurashi, Amin Zagzoog, Fahad Almehmadi, Faisal Al-Husayni, Akram Ahmad, Saad Albugami

**Affiliations:** 1 Department of Cardiac Sciences, King Faisal Cardiac Center, National Guard Hospital, King Abdulaziz Medical City, Jeddah, SAU; 2 Department of Cardiac Sciences, King Abdullah International Medical Research Center, King Abdulaziz Medical City, Jeddah, SAU; 3 Department of Cardiac Sciences, King Saud bin Abdulaziz University for Health Sciences, King Abdulaziz Medical City, Jeddah, SAU; 4 Department of Internal Medicine, King Faisal Cardiac Center, National Guard Hospital, King Abdulaziz Medical City, Jeddah, SAU; 5 Department of Cardiology, Dr. Samir Abbas Hospital, Jeddah, SAU

**Keywords:** angina in the young, unroofing procedure, life-threatening arrhythmia, anomalous coronary artery origin, ventricular arrhythmia, malignant arrhythmia, anomalous origin of right coronary artery, anomalous rca

## Abstract

Anomalous aortic origin of a coronary artery (AAOCA) is a congenital condition that can lead to sudden cardiac death (SCD), particularly among young individuals. The cause of SCD is thought to be ischemia, primarily related to the course of the anomalous coronary artery. Surgical intervention, such as unroofing or coronary revascularization, is the preferred management modality for patients with evidence of ischemia or concomitant fixed obstruction. Herein, we presented a case of a 24-year-old male admitted to the emergency department with a history of palpitations, dyspnea, diaphoresis, and syncope. The patient had no prior medical diseases and was eventually diagnosed with an anomalous right coronary artery (ARCA) originating from the left coronary sinus. The patient underwent surgical unroofing of the ARCA to prevent further episodes of ischemia and ventricular arrhythmias. The case highlights that coronary artery anomalies can be life-threatening and lead to SCD, especially in young individuals with no risk factors. Investigating coronary anomalies in medically free patients presenting with cardiac symptoms and arrhythmias is crucial.

## Introduction

Anomalous aortic origin of a coronary artery (AAOCA) is one of the nonatherosclerotic coronary artery abnormalities known to cause sudden cardiac death (SCD), especially in young individuals [1]. It is congenitally acquired, and the mechanism of SCD is thought to be ischemia mediated and is primarily related to the course of the anomalous coronary artery, with the inter-arterial course being associated with worse outcomes [2]. The prevalence is estimated to be somewhere between 0.2% and 2.0% on invasive and non-invasive cardiac testing [3]. The preferred management modality, when there is evidence of ischemia, is a surgical intervention that involves unroofing or coronary revascularization for patients with concomitant fixed obstruction [4].

## Case presentation

**Figure 1:**
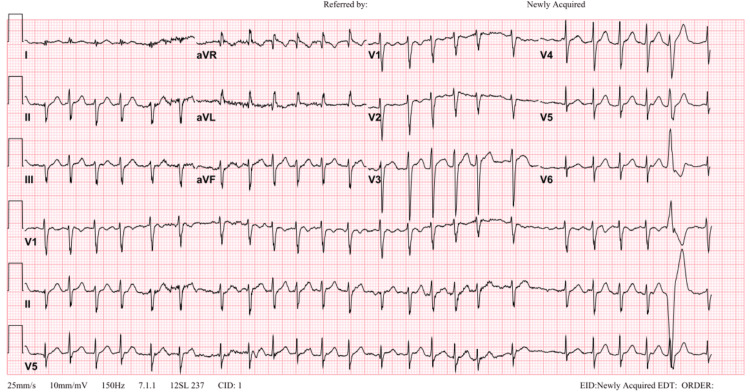
Initial ECG showing AFib with rapid ventricular response

We describe a case of a 24-year-old male who was not previously known to have any medical illness and presented to the emergency department with a history of palpitations, dyspnea, diaphoresis, and an episode of syncope. The patient denied taking any medication, illicit drugs, or consuming alcohol. Upon initial evaluation, he had irregular tachycardia with normal blood pressure, and the initial ECG was interpreted as atrial fibrillation (AFib) with rapid ventricular response (Figure 1).

**Figure 2:**
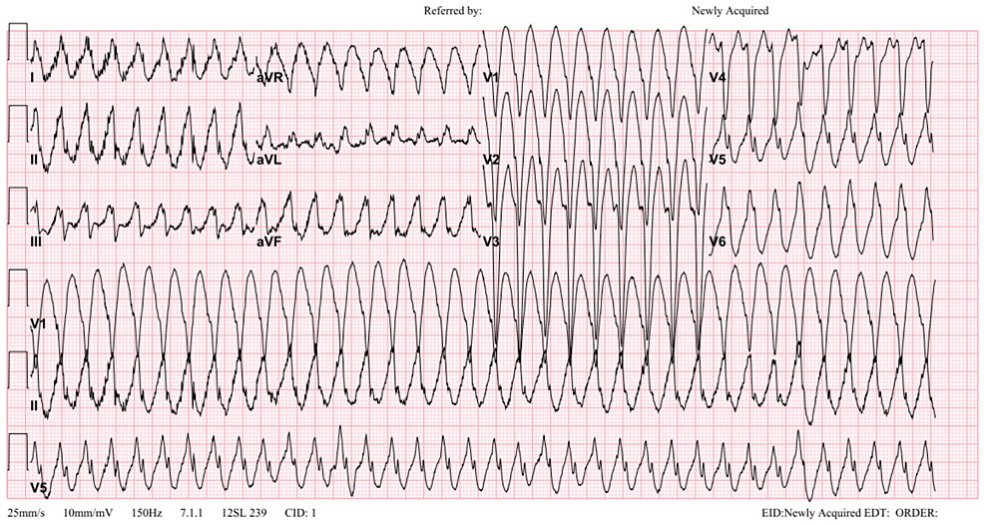
Patient’s ECG showing VT

During the physical examination, the patient developed chest pain, decreased level of consciousness, and wide complex tachycardia, interpreted as ventricular tachycardia (VT) (Figure 2), which prompted sedation and synchronized cardioversion.

**Figure 3:**
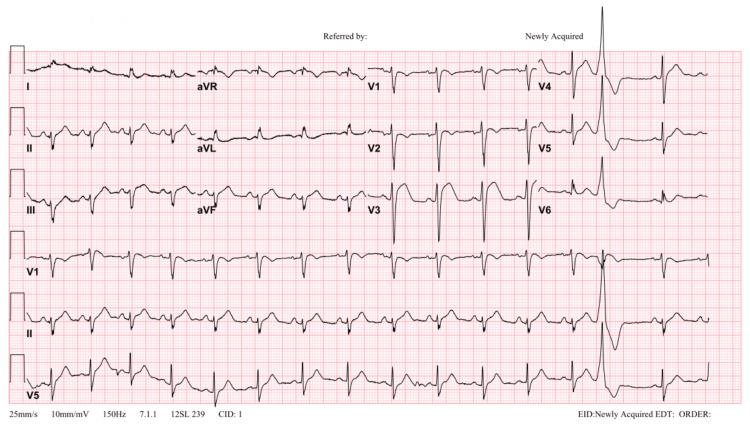
Patient’ ECG revealing an ST-segment elevation in the anterior leads

Post-cardioversion, his rhythm converted to polymorphic VT. He achieved a return of spontaneous circulation after intravenous amiodarone and cardiopulmonary resuscitation for one minute. Post-recovery, ECG showed sinus rhythm with ST elevation involving anterior precordial leads (Figure 3). The patient’s lab results showed a normal complete blood count, electrolytes, renal function, coagulation profile, and liver profile. His troponin level was 157 pg/mL (reference <34.20 pg/mL).

Urgent coronary angiography revealed patent epicardial coronary arteries, but the right coronary artery (RCA) had an anomalous origin from the left coronary sinus, also called anomalous right coronary artery (ARCA) (Video 1).

**Video 1:** Coronary angiogram showing the RCA with an anomalous origin from the left coronary sinus

**Figure 4:**
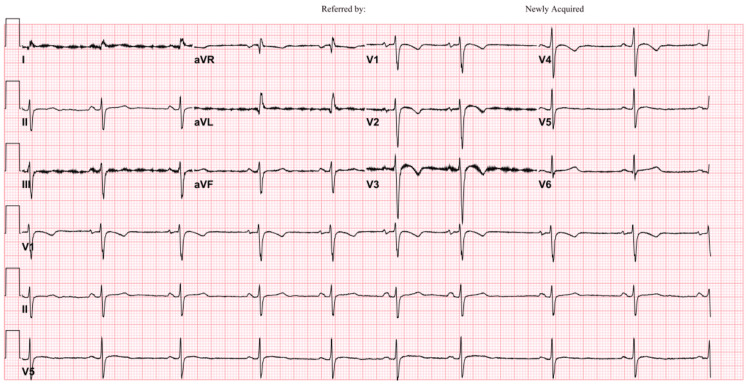
Follow-up ECG demonstrating sinus bradycardia with left anterior fascicular block

Follow-up ECG showed sinus bradycardia with a left anterior fascicular block, resolving ST elevation and T wave inversion (Figure 4).

The patient was treated with amiodarone, metoprolol succinate, and apixaban.

**Figure 5:**
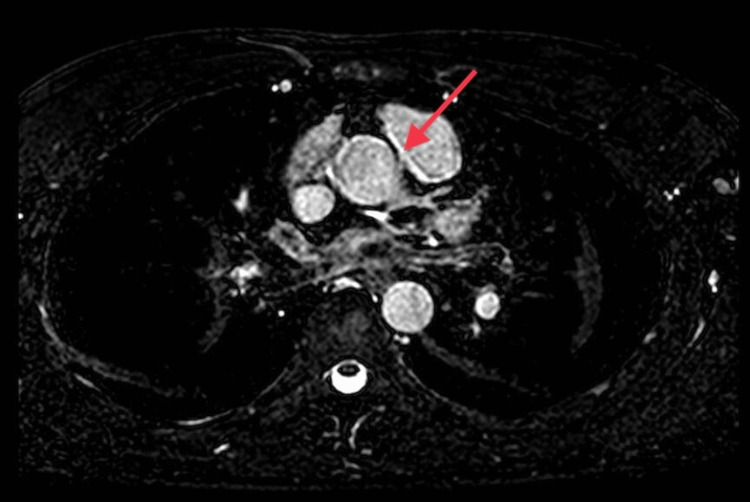
CMR imaging of the ARCA (red arrow) originating from the left coronary cusp

**Figure 6:**
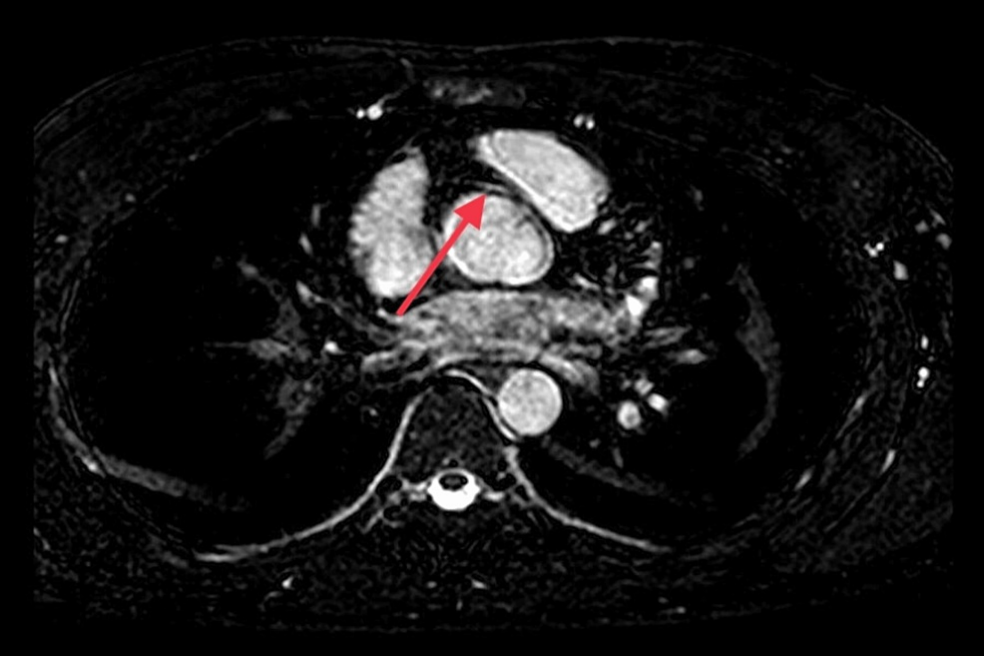
CMR imaging demonstrating an interarterial course of the RCA (red arrow) between the aorta and pulmonary artery

Transthoracic echocardiography showed normal left ventricle (LV) size, systolic function, and no resting regional wall motion abnormality. To further define the origin and course of the anomalous coronary artery, cardiac magnetic resonance (CMR) imaging showed that the RCA is originating from the anterior aspect of the left coronary cusp from a slit-like opening (Figure 5), an intramural course of the proximal RCA, coursing between the aorta and pulmonary artery (Figure 6) with diffuse narrowing of the lumen of around 30%.

CMR also showed normal LV size, function, and no delayed gadolinium enhancement.

The patient underwent surgical unroofing of the ARCA to prevent further episodes of ischemia and ventricular arrhythmias. He remained asymptomatic for two years after surgical repair and had multiple normal Holter studies.

## Discussion

Coronary artery anomalies (CAAs) refer to a group of congenital disorders where one of the three primary epicardial coronary arteries has an abnormal origin or path [5,6]. CAAs are uncommon and can be generally categorized as abnormalities of the origin, course, destination, size, or number of vessels in the coronary arteries. Classification is usually based on the ectopic anomalous coronary artery origin, which may arise from the aorta, the pulmonary artery, an incorrect sinus valsalva, or a branch from a different coronary artery [7,8]. The term CAA has historically been used to denote cases that affect less than 1% of the general population.

One study has found that the overall incidence of CAAs was 5.64% [9]. Another study found that a coronary artery with anomalous origin is an unusual occurrence with a 0.26% prevalence rate [10]. This significant variability in the observed prevalence of AAOCA is attributed to inherent referral bias.

RCA abnormality makes up about 8% of all coronary anomalies, of which the origin of RCA from the left coronary sinus has an incidence of 0.03% to 0.11% [11]. The interarterial RCA anomaly frequency is 0.23%. However, the true prevalence of AAOCA in the general population remains unknown [10].

These congenital artery anomalies could lead to SCD, making them a serious life-threatening condition, especially in young individuals engaged in sports. In young athletes, AAOCA is considered the second leading cause of SCD [12]. The prevalence of AAOCA and their associated absolute risk of SCD in the general population is unknown. This created controversy regarding the optimal approach to risk stratification and management of this population.

Several pathophysiological mechanisms that predispose individuals to SCD in those with AAOCA have been postulated. In summary, when current knowledge and persisting uncertainties are balanced, ischemia remains the most likely mechanism of SCD. Commonly, it was thought that the interarterial course might be associated with an increased risk of ischemia due to compression of the anomalous coronary between the great vessels [13]. Another proposed mechanism is that these anomalies usually arise at an acute or high takeoff angle, which is prone to the systolic expansion of the aorta, causing kinking or precipitating coronary spasms [3]. Recently, a new mechanism suggested that the existence of an initial intramural course may be pivotal in the pathogenesis of ischemia. Severe functional stenosis is intrinsically related to the intramural course that results in varying degrees of lateral compression secondary to the shear stress exerted by the aortic walls, particularly in the presence of increased aortic stiffness [14,15]. Despite multiple theories, the precise causes of ischemia and sudden cardiac events are probably multifactorial. Thus, risk stratification has proven difficult, and surgical indications remain unclear.

Most patients do not exhibit symptoms or only experience them after strenuous physical activity. Nonetheless, patients can present with angina, myocardial infarction, syncope, arrhythmia, or sudden cardiac arrest [16,17]. The onset of symptoms is unpredictable, and the first symptom may be SCD. The timing of symptoms onset is unpredictable, and the first symptom may be SCD [18]. Various diagnostic tests can be used to assess coronary artery abnormalities, including transesophageal echocardiography, magnetic resonance angiography, coronary computed tomography angiography (CCTA), and invasive coronary angiography (ICA) [19]. According to the American College of Cardiology and American Heart Association (ACC/AHA) 2018 Guidelines for managing adults with congenital heart disease, there is no universal consensus regarding assessing CAAs [4]. ICA was considered the most important and definite tool to identify and classify CAA. However, CCTA is currently considered the gold standard due to a characterization of the anatomic clues associated with high-risk CAAs and allows visualization of the surrounding cardiac structures and their relative three-dimensional relations [20]. Current AHA guidelines suggest using a provocative test, but no standardized protocols have been proposed to stratify CAA-related ischemia because there are controversial data regarding its validity [4,13].

Anomalous aortic origins of coronaries’ prognostic consequences are extremely fluctuating. Generally, it is believed that the presence of a lone ARCA that originates from the left sinus of Valsalva (ACAOS) with interarterial course had benign clinical courses [21]. Nevertheless, our case demonstrates that the right ACAOS can have a lethal prognosis. Thus, it is necessary to identify high-risk features in patients with ACAOS to establish screening protocols to prevent such an outcome. Furthermore, any young patient presenting with ventricular arrhythmias or angina without cardiac risk factors should be considered for coronary anomalies [22].

## Conclusions

CAAs are a group of congenital disorders that can affect the anatomy or number of vessels in the coronary arteries. Although CAAs are uncommon, they can be life-threatening and contribute to SCD. We presented a case of a young patient presenting with angina and ventricular arrhythmias without cardiac risk factors. His investigation revealed RCA which is originating from the anterior aspect of the left coronary cusp with a course between the aorta and pulmonary artery. Such a case warrants investigating coronary anomalies in patients presenting with cardiac symptoms and arrhythmias without risk factors. Further studies may also be beneficial to risk-stratify such cases and describe the management modality.
